# Lifting the mask mandate puts our priorities in plain sight

**DOI:** 10.1002/jhm.12909

**Published:** 2022-07-07

**Authors:** Tara Calhoun, Ndidi Unaka

**Affiliations:** ^1^ Division of Hospital Medicine, Cincinnati Children's Hospital Medical Center Cincinnati Ohio USA; ^2^ Department of Pediatrics University of Cincinnati College of Medicine Cincinnati Ohio USA

## INTRODUCTION

In January 2021, the Biden administration issued an executive order that required individuals to wear face masks when using public transportation, including airplanes, trains, city buses, and ride‐share vehicles to “save lives and allow all Americans, including the millions of people employed in the transportation industry, to travel and work safely.”^1^ On April 18, 2022, a federal judge in Florida ruled that the Centers for Disease Control and Prevention (CDC) could no longer enforce the mask mandate. As a result, individuals were permitted to use their discretion regarding the use of masks while traveling on any form of public transportation. The ruling calls into question the authority of the CDC to make decisions regarding public health protections, which may have far‐reaching implications.^2^ This mandate was overturned at a time when only 66% of the US population were fully vaccinated against coronavirus disease 2019 (COVID‐19) and approximately 18 million children under 5 years of age were ineligible to receive the vaccine.^3^, ^4^ The discontinuation of the mask mandate places marginalized populations—including those employed in the transportation industry as well as those who primarily depend on public transportation for their daily commute—at higher risk for COVID‐19 infection. Finally, the ruling minimizes the devastating impact of the pandemic in the United States and ignores the significant surges in cases, hospitalizations, and deaths associated with COVID‐19 variants that could continue to plague us for months or years to come.

## LIFTING THE MASK MANDATE LEAVES CHILDREN EXPOSED

We cannot ignore the deleterious effects of the COVID‐19 pandemic on the physical, mental, and social health and well‐being of children. Children remain one of the most susceptible populations to COVID‐19 infection and can be asymptotic carriers of severe acute respiratory syndrome coronavirus 2 (SARS‐CoV‐2). Although the COVID‐19 vaccine is approved for children 5 years and older, only 28.7% of children aged 5–11 years are fully vaccinated; children 0–4 years of age remain ineligible for the vaccine.^3^ That leaves a substantial percentage of children who are at risk of developing severe disease. During the Omicron surge, COVID‐19‐associated hospitalization rates among children 5–11 years of age were 1.7 times higher among those who were unvaccinated compared to vaccinated children. Nearly one‐third of hospitalized children had no underlying medical conditions, and 19% required intensive care.^5^ Similarly, the peak hospitalization rate for children aged 0–4 years during the Omicron surge was approximately five times greater than the peak hospitalization rate during the Delta surge.^6^ Additionally, we continue to bear witness to the mental health crisis in children, which has only been exacerbated during the pandemic; the factors contributing to this crisis are numerous and in many instances interconnected. School closures followed by the vacillation between virtual and in‐person instruction, social isolation, mounting economic stressors on families, and limited access to mental health providers are just a few of the factors at play.^7^ Additionally, we watch in anguish as children experience the trauma resulting from the loss of parents and caregivers who have died from COVID‐19; in fact, over 140,000 children have lost a custodial parent or caregiver and 65% of those affected are children of racial and ethnic minorities.^8^

Donning a face mask decreases person‐to‐person transmission of SARS‐CoV‐2 and other viruses by 50%–80%, which underscores the importance of its usage by individuals on public modes of transportation.^9^, ^10^, ^11^ This is particularly relevant given that a significant proportion of children and adolescents depend on public transportation to get to and from school. Although yellow school buses do not fall under the public transportation mask mandate, it highlights an example of the recurrent risk of COVID‐19 exposure given that children who ride the bus are in close proximity to one another in a confined space. In 2016, approximately 20 million students 5–14 years of age traveled over 2 miles to attend school. Approximately 60% of students from low‐income, vehicle‐owning families depend on the yellow bus to get to school; 70% of children from low‐income families without a vehicle take the bus to school.^12^ In Cincinnati, Ohio, 14,500 public high school students rely on city buses to attend school and/or work.^13^ In Baltimore, Maryland, 6 out of 10 high school students commute on the Metro or light rail, which represents roughly 13%–16% of all weekday commuters in the Maryland Transit Administration system.^14^

## THE IMPACT OF LIFTING THE MASKING MANDATE ON MARGINALIZED COMMUNITIES

The disproportionate impact of COVID‐19 on historically marginalized communities cannot be overstated. The demographics of the public transportation workforce as well as the existing disparities in the dependent use of public transportation among the poor and marginalized communities of color that exist in the United States illustrate the direct link between systems rooted in structural racism and increased COVID‐19 infection risk and its consequences. While only 5% (7.8 million) of US workers rely on public transportation, public transit usage varies greatly by region and sociodemographic factors. Among US workers, those who identify as Black and Hispanic were more likely to be public transportation commuters. When analyzing public transportation use outside of transit‐heavy metropolitan areas like New York and San Francisco, approximately 44% of public transportation commuters had an annual income of less than $25,000.^15^ Moreover, those who are employed by transportation entities are disproportionately Black, Hispanic, and/or in the low‐income bracket; in fact, census data in 2016 showed that 27.3% of bus drivers in the United States self‐identified as Black. And, this group of essential workers have not been spared from the perils of this pandemic—in New York City alone, 136 employees of Metro Transit Authority died from COVID‐19 between March 2020 and January 2021 and greater than half of those were employees of color.^16^, ^17^

## UNMASKING AND AIR TRANSPORTATION

The COVID‐19 pandemic has taken its toll on the air transportation industry. The emergence of the pandemic was associated with flight suspensions and a precipitous drop in flight sales. Flight attendants were thrust into the roles of referees as they attempted to quell on‐flight quarrels and were subjected to harassment and abuse by customers who refused to wear masks. As a result, airlines were strong proponents for the lifting of the mask mandate. While accurately tracking transmission rates resulting from air travel is challenging, airports and airplanes pose large‐scale, unequivocal infection risks to passengers and employees alike. Air travel may drive the transmission and spread of COVID‐19 through (1) the gathering of large crowds in airports and (2) in‐flight exposure to COVID‐19, resulting from passengers in enclosed spaces for variable periods of time. Airlines have been tested in their ability to effectively promote safety, efficiency, and customer experience while facing the realities of negative profit margins. However, the industry's support of the lifting of the mask mandate leaves us pondering if their advocacy took into account the potential human cost in addition to their bottom line.

## THE PRICE OF UNMASKING AND A CALL TO ACTION

The federal court ruling to lift the mask mandate on public transportation undermines the authority of the CDC and allows the COVID‐19 pandemic to threaten more lives. In the United States alone, approximately one million people have died as a result of COVID‐19 infection.^7^ While we have experienced intermittent decreases in COVID‐19 cases and hospitalizations, the pandemic is far from over. The mortality rate of COVID‐19 is approximately 10 times greater than the mortality rate for other common viruses like influenza and respiratory syncytial virus.^18^ Since April 2022, the 7‐day moving average of COVID‐19‐related cases has increased by approximately 20% and an average of 280 people in the United States continue to die from COVID‐19 infection each week.^3^ In addition, the robust use of at‐home COVID‐19 tests has contributed to significant underreporting of COVID‐19 cases and associated challenges monitoring “hot spots” or exposure risk. Rates of COVID‐19 cases are estimated to be 14.5 times higher than what is currently reported, which may falsely reassure the public.^19^ Thus, the transition to a “new normal”—a state that mirrors our approach to other common respiratory viruses—is contingent upon a decrease in the virulence of COVID‐19 in conjunction with a continued increase in vaccination rates and immunity among the population.^18^ Given the unpredictable nature of the pandemic, the timing at which we will transition to this state of being remains uncertain.

Time after time, we compromise the health and well‐being of those who depend on us the most. A safe and equitable transportation system must incorporate provisions to protect everyone in it, including commuters and employees. We grapple with the COVID‐19 pandemic and the diseases of racism and inequity—these ailments have worked in tandem to mar our communities and deplete our potential. We must drown out the noise of those who decry the commonsense use of masks. Government officials and companies must develop an understanding of the fragility of public trust, which destabilizes with every fluctuating recommendation and contradiction. We must fully engage in the battle against science and disinformation if we ever want to win the war. Finally, we need to move away from a culture of individualism to one that is a champion for the common good.

## CONFLICTS OF INTEREST

The authors declare no conflicts of interest.
